# Supplementary figures and images for: Feeling tired versus feeling relaxed: Two faces of low physiological arousal
Source: PLoS One. 2024 Sep 9;19(9):e0310034. doi: 10.1371/journal.pone.0310034 (PMC11383234; doi:10.1371/journal.pone.0310034)

Standard Error

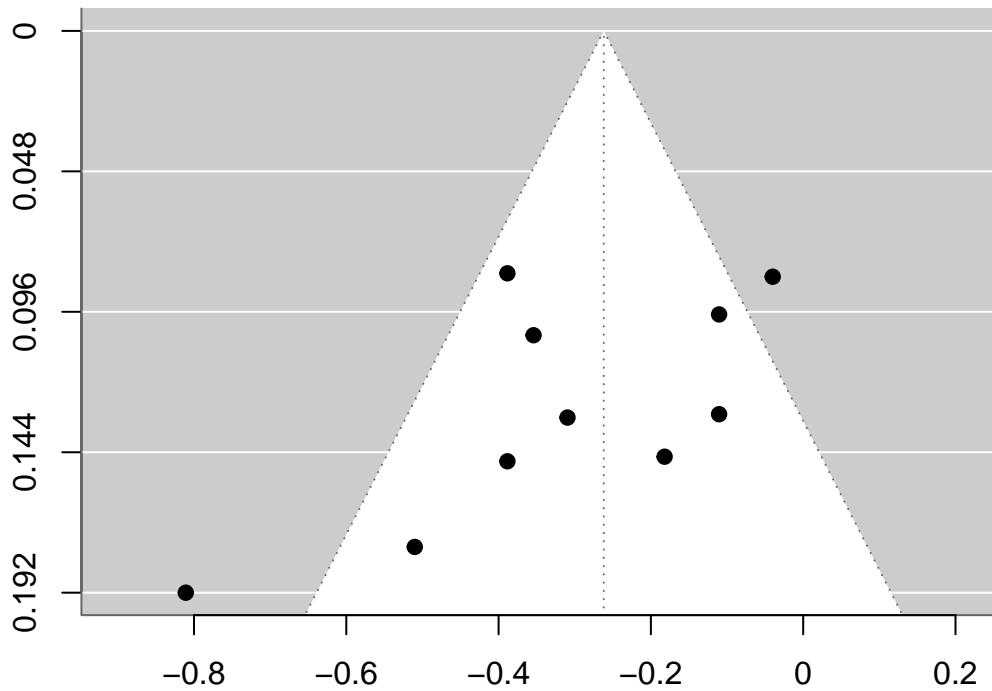

Fisher's z Transformed Correlation Coefficient

Supplement: S1 Fig — Funnel Plot of the used datasets. Each point plotted represents the weighted z-transformed correlation of one dataset described in Table 2. The vertical dotted line represents the estimated correlation, as reported in Fig 1. The white triangle represents the region in which 95% of the data points should lie in absence of a selection bias. (PDF) [file pone.0310034.s001.pdf]

Standard Error

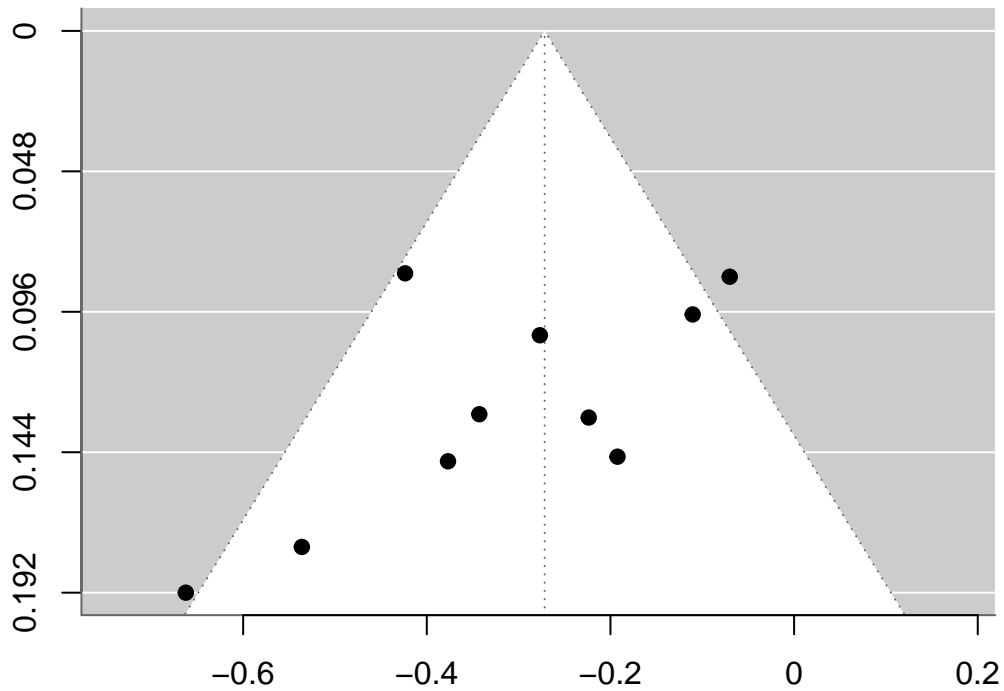

Fisher's z Transformed Correlation Coefficient

Supplement: S2 Fig — Funnel Plot of the used datasets. Each point plotted represents the weighted z-transformed correlation of one dataset described in Table 2. The vertical dotted line represents the estimated correlation, as reported in Fig 2. The white triangle represents the region in which 95% of the data points should lie in absence of a selection bias. (PDF) [file pone.0310034.s002.pdf]

Standard Error

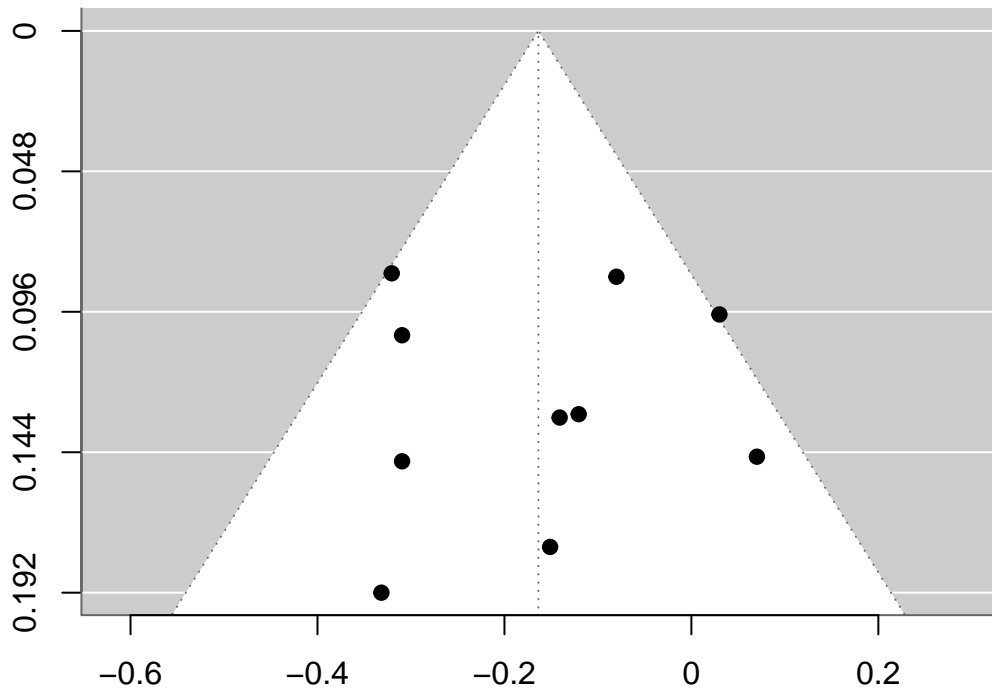

Fisher's z Transformed Correlation Coefficient

Supplement: S3 Fig — Funnel Plot of the used datasets. Each point plotted represents the weighted z-transformed correlation of one dataset described in Table 2. The vertical dotted line represents the estimated correlation, as reported in Fig 3. The white triangle represents the region in which 95% of the data points should lie in absence of a selection bias. (PDF) [file pone.0310034.s003.pdf]

Standard Error

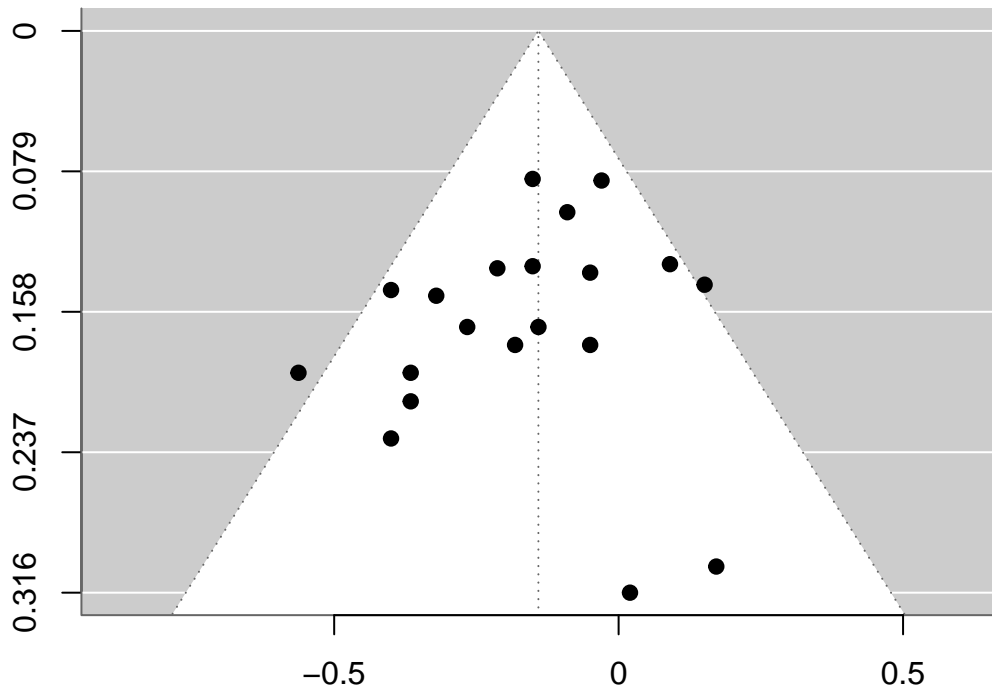

Fisher's z Transformed Correlation Coefficient

Supplement: S4 Fig — Funnel Plot of the used datasets. Each point plotted represents the weighted z-transformed correlation of one dataset described in Table 2. The vertical dotted line represents the estimated correlation, as reported in Fig 4. The white triangle represents the region in which 95% of the data points should lie in absence of a selection bias. (PDF) [file pone.0310034.s004.pdf]

Standard Error

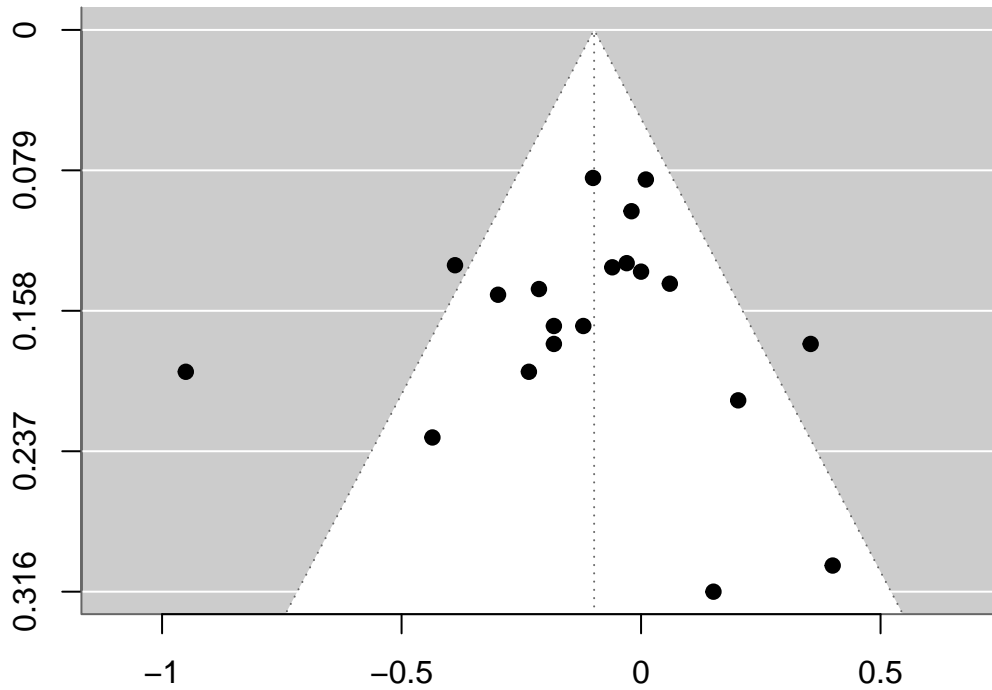

Fisher's z Transformed Correlation Coefficient

Supplement: S5 Fig — Funnel Plot of the used datasets. Each point plotted represents the weighted z-transformed correlation of one dataset described in Table 2. The vertical dotted line represents the estimated correlation, as reported in Fig 5. The white triangle represents the region in which 95% of the data points should lie in absence of a selection bias. (PDF) [file pone.0310034.s005.pdf]

Standard Error

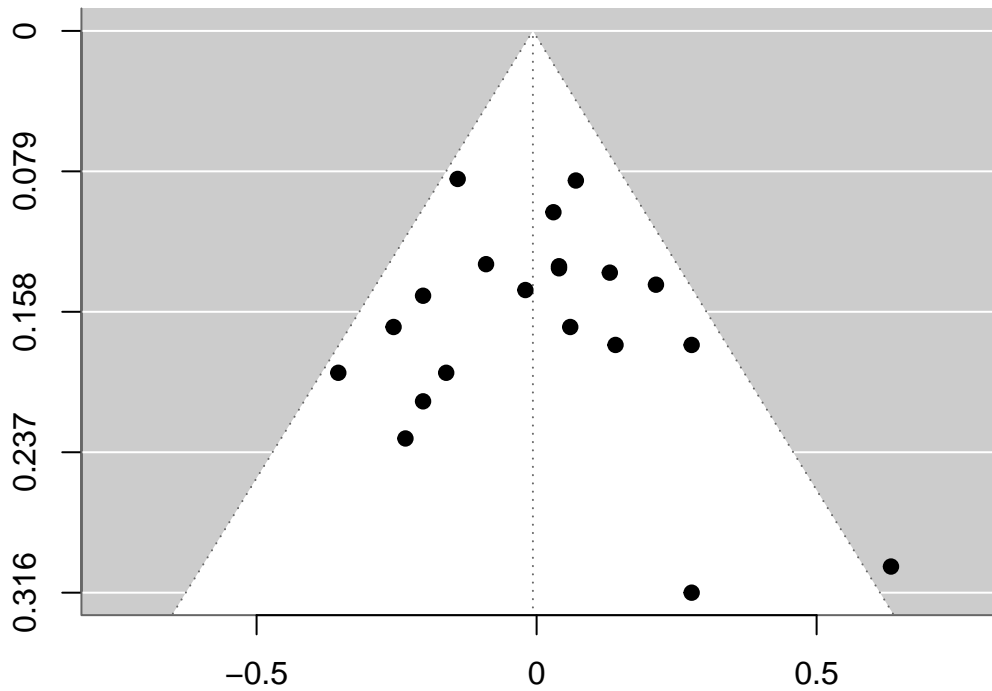

Fisher's z Transformed Correlation Coefficient

Supplement: S6 Fig — Funnel Plot of the used datasets. Each point plotted represents the weighted z-transformed correlation of one dataset described in Table 2. The vertical dotted line represents the estimated correlation, as reported in Fig 6. The white triangle represents the region in which 95% of the data points should lie in absence of a selection bias. (PDF) [file pone.0310034.s006.pdf]
