# Supplementary material for: Feeling tired versus feeling relaxed: Two faces of low physiological arousal
Source: PLoS One. 2024 Sep 9;19(9):e0310034. doi: 10.1371/journal.pone.0310034 (PMC11383234; doi:10.1371/journal.pone.0310034)
Supplement: S1 Table — n = number of studies in this category; p values in bold indicate significance. (PDF) [file pone.0310034.s007.pdf]

	Post-intervention			
	relaxing (n = 9) *M* (*SD*)	non relaxing (n = 11) *M* (*SD*)	*p*	BF_{10}
Muscle Scale	-.11 (.19)	-.2 (.19)	.34	0.56
General Relaxation Scale	-.01 (.19)	-.16 (.3)	.18	0.74
Cardio-vascular Scale	.04 (.26)	-.01 (.19)	.641	0.43
