# Supplementary material for: Feeling tired versus feeling relaxed: Two faces of low physiological arousal
Source: PLoS One. 2024 Sep 9;19(9):e0310034. doi: 10.1371/journal.pone.0310034 (PMC11383234; doi:10.1371/journal.pone.0310034)
Supplement: S2 Table — n = number of studies in this category; p values in bold indicate significance. (PDF) [file pone.0310034.s008.pdf]

	Pre-intervention				Post-intervention			
	rep. measure (n = 4) *M* (*SD*)	no rep. measure (n = 6) *M* (*SD*)	*p*	BF_{10}	rep. measure (n = 9) *M* (*SD*)	no rep. measure (n = 11) *M* (*SD*)	*p*	BF_{10}
Muscle Scale	-.41 (.21)	-.22 (.15)	.2	1	-.23 (.14)	-.1 (.21)	.107	0.99
General Relaxation Scale	-.37 (.19)	-.26 (.14)	.358	0.71	-.2 (.29)	0 (.21)	.105	1.16
Cardio-vascular Scale	-.14 (.16)	-.18 (.14)	.666	0.53	-.02 (.18)	.03 (.25)	.632	0.43
